# A study on Korean nursing students' educational outcomes

**DOI:** 10.3352/jeehp.2011.8.3

**Published:** 2011-04-04

**Authors:** Kasil Oh, Yang Heui Ahn, Hyang-Yeon Lee, Sook-Ja Lee, In-Ja Kim, Kyung-Sook Choi, Myung-Sook Ko

**Affiliations:** 1 School of Nursing, Ulaanbaartar University, Mongolia.; 2 Department of Nursing, Yonsei University Wonju College of Medicine, Wonju, Korea.; 3 College of Nursing, Kyung Hee University, Seoul, Korea.; 4 College of Nursing, Korea University, Seoul, Korea.; 5 Department of Nursing, Daejeon University, Daejeon, Korea.; 6 Department of Nursing, Chung-Ang University, Seoul, Korea.; 7 Christian College of Nursing, Gwangju, Korea.

**Keywords:** Education, Outcomes, Korea, Nursing students

## Abstract

The purpose of this study was to describe outcome indicators of nursing education including critical thinking, professionalism, leadership, and communication and to evaluate differences among nursing programs and academic years. A descriptive research design was employed. A total of 454 students from four year baccalaureate (BS) nursing programs and two three-year associate degree (AD) programs consented to complete self-administered questionnaires. The variables were critical thinking, professionalism, leadership and communication. Descriptive statistics, χ^2^-test, t-tests, ANOVA, and the Tukey test were utilized for the data analysis. All the mean scores of the variables were above average for the test instruments utilized. Among the BS students, those in the upper classes tended to attain higher scores, but this tendency was not identified in AD students. There were significant differences between BS students and AD students for the mean scores of leadership and communication. These findings suggested the need for further research to define properties of nursing educational outcomes, and to develop standardized instruments for research replication and verification.

## INTRODUCTION

Nursing education aims to have students acquire the knowledge, practical skills, and social responsibility necessary to thoroughly assume their role as professional nurses after completing a nursing program [1]. Educational outcomes are statements of the professional abilities that nursing students should achieve during undergraduate study. Outcome statements incorporate philosophical approaches, indicate core curriculum concepts, and describe key professional abilities in a comprehensive, holistic fashion [2].

Since the Korean Council for University Education (KCUE) introduced nursing education accreditation in 1997, arguments have been raised both inside and outside of the circles concerned. Without devising objective measuring tools to satisfy the parties involved, the accreditation system will not be valid in real terms. Meanwhile, the KCUE tried to adopt evaluation criteria applicable to all educational institutions including nursing schools, but it found that more adaptable evaluation criteria for nursing schools would be necessary. The Korean Accreditation Board of Nursing (KABON) was established in 2003 under such circumstances as accreditation problems. In 2006, KABON revised the standards and criteria for nursing program based on the studies by Ahn et al. [3] and Kim et al. [1], which identified critical thinking, professionalism, leadership, communication, skills for nursing practice, and humanity as core competencies of nursing education. These competencies were parallel to Lenburg's suggested essential core competencies for students [4]. These included assessment and intervention, communication, critical thinking, teaching, human caring relationships, management, leadership, and knowledge integration skills, which are found in the Competency Outcomes and Performance assessment (COPA) Model. Further, in the US, after already having claimed to consider its professionals characteristics in the accreditation system of the fields of public health and medicine [5], in 2002 the National League for Nursing Accrediting Commission (NLNAC), added critical thinking, communication, and curative nursing intervention as a measuring province of educational outcomes. Recently NLNAC [6] explained those as student learning outcomes. Nevertheless, few studies describing the educational outcomes of core competencies have been found in the nursing literature. Based on the previously noted literature KABON core competencies, four cores - critical thinking, professionalism, leadership, and communication - were selected as primary nursing outcome indicators, to evaluate nursing students.

This study aims to describe the critical thinking, professionalism, leadership, and communication of nursing students, and to compare the differences in critical thinking, professionalism, leadership, and communication between types of nursing programs and among academic years.

## METHODS

### Research design

A descriptive research design was employed for evaluating critical thinking, professionalism, leadership, and communication of nursing students.

### Research subjects

This study targeted nursing students from three-year associate degree (AD) programs and baccalaureate (BS) programs nationwide. Selection criteria for its subjects were as described below. First, considering the regions where nursing schools were accessible, four BS programs and two three-year AD programs located in Seoul, Gangwon, Choongcheong, Jeonra, and Kyungsang Provinces were selected. Second, 15-16 students per each year in school in BS programs and 34-35 per each year in school in three-year AD programs were sampled by convenience. The total number of participants was 530 students, including 320 from the BS programs and 210 from the three-year AD programs. Of these, 454 subjects were included in the final data analysis, after incomplete surveys were eliminated.

### Ethical considerations and human participant protection

Taking the ethical aspect of the procedure into account, subjects were informed of the purpose of the study and the right to cancel their participation even during the response process. They were informed there would be no penalties for canceling their participation. Subjects agreed to the purpose of this study, voluntarily signed a form, and participated in the survey.

### Research instruments

The research instruments used in this study were as follows:

1) Critical Thinking Disposition Instrument (CTDI): The CTDI, developed by Yoon [7], was used for measuring critical thinking disposition. Skills in critical thinking can provide the creative solutions and multiple pathways required for successful quality-improvement initiatives. CTD describes attributes or habits of the mind integrated into an individual's beliefs or actions that are conducive to critical thinking skills (CTS). The CTD instrument measures intellectual eagerness/curiosity, prudence, self-confidence, systemicity, intellectual fairness, healthy skepticism, and objectivity. Each item is scored on a five-point Likert scale. The range of scores is from 27-135 with higher scores indicating higher levels of critical thinking disposition. The reliability of the instrument was Cronbach's alpha 0.89 in the original study [7]. The reliability of the instrument in this study was Cronbach's alpha 0.88.

2) Nurse Self-Description Form (NSDF): The NSDF, developed by Dagenais and Meleis [8], was used for measuring professionalism. It was validated for nurses and nursing students by the Western Council on Higher Education for Nursing (WCHEN) researchers in a study of the effects of a leadership program in predicting nursing performance [8], and it has demonstrated validity when used to study productivity of RN-BSN students in Korea [9]. Each of the 19 items of the Nurse Self-Description Form-Korean version is scored on a five-point Likert scale. The range of scores is from 19-95 with higher scores indicating higher levels of professionalism. The reliability of the instrument was Cronbach's alpha 0.93 in the original study [8]. Cronbach's alpha was 0.92 in the study of Kim et al. [9]. The reliability of the instrument in this study was Cronbach's alpha 0.88.

3) Self-Assessment Leadership Instrument (SALI): The SALI was developed by Smola [10], and measures leadership behavior and characteristics in baccalaureate nursing students. The 40-item instrument relies on self assessment of critical thinking and decision making skills, interpersonal relationships, group relations, and job relations. Each item is scored on a five-point Likert scale. The range of scores is from 40-200, with higher scores indicating increased occurrence of leadership behavior. The reliability of the instrument was Cohen's K coefficient of 0.545 in the original study [10]. Cronbach's alpha was 0.95 in the study of Oh et al. [11]. The reliability of the instrument in this study was Cronbach's alpha 0.94.

4) Supportive Communication Inventory (SCI): The SCI, developed by Whetten and Cameron [12], was used to assess communication performance for students who majored in management. Supportive communication is communication that seeks to preserve a positive relationship between the communicators while still addressing the problem at hand. Supportive communication has eight attributes, which include being problem-oriented, congruent, descriptive, validating, specific, conjunctive, owned, and committed to supportive listening [12]. Each of the 20 items is assigned a score with the range from one (never agree) to six (positively agree). The range of scores is from 20 to 120, with higher scores indicating higher communicative skills. The reliability of the instrument was Cronbach's alpha 0.86 in study by Oh et al. [11]. The reliability of the instrument in this study was Cronbach's alpha 0.94.

### Data collection procedure

One student per year in five BS programs and one three-year AD program was sampled by convenience, resulting in a total of 23 students in the preliminary survey. Since there were no unusual matters concerning the fitness of questions, time, or procedure, the original questionnaires were used without revision. The data collection involved using self-administered questionnaires in the main survey. The researchers directly distributed the questionnaires to the students and gathered their responses. It took the students about 20-25 minutes to complete the questionnaires.

### Data analysis

Data were analyzed with SPSS ver. 12.0 (SPSS Inc., Chicago, IL, USA). Descriptive statistics were utilized to evaluate general characteristics of the subjects.

χ^2^-test, t-tests, ANOVA, and the Tukey test were utilized to assess differences among nursing programs and academic years.

Cronbach's alpha coefficients were utilized to evaluate the reliability of the instruments.

## RESULTS

### General characteristics of the subjects

General characteristics of the 246 (54.2%) BS students and 208 (45.8%) AD students are shown in Table 1. Regarding BS students, the average age of the students was 21.2 (±1.82) years, and most of them were female (94.2%) and single (98.8%). There were 58 (23.6%) in the first year, 62 (25.2%) in the second, 63 (25.6%) in the third, and 63 (25.6%) in the fourth. Likewise the average age of AD students was 21.5 (±2.54) years, and most of them were female (90.9%) and single (98.1%). There were 58 (27.9%) in the first year, 84 (40.4%) in the second, 66 (31.7%) in the third. There were no differences in age or marital status (P>0.05). The only difference between the BS and AD students was the gender distribution (χ^2^=8.069, P=0. 007).

### Critical thinking, professionalism, leadership, and communication of nursing students

The critical thinking, professionalism, leadership, and communication mean scores of the students are as shown Table 2. The average score for critical thinking was 85.3 out of a possible of 135, and the scores tended to be higher for students who had completed more years of education. Scores for seniors were the highest with an average of 88.2. The average scores for juniors and sophomores were 85.8 and 84.4, respectively. The average score for professionalism was 68.2 out of a total possible of 95. The mean score of seniors was the highest at 70.7, while the score of the freshmen was higher than the scores for sophomores and juniors. The average score for leadership was 149.2 out of a total possible of 200, and the mean score of seniors was the highest as 155. 3, while the score for freshmen was higher than the scores for sophomores and juniors. The average score for communication was 85.2 out of a possible of 120, with increasing scores accompanying more years of education. Senior scores were the highest with an average of 86.2. Junior and sophomore scores were 85.7 and 84.8, respectively.

All indicators of critical thinking, professionalism, leadership, and communication measured a little higher than average scores for these testing instruments. Results from Tukey's post hoc test showed that the mean score of seniors were significantly higher when compared to freshmen, sophomores, and juniors in all variables except for communication (Table 2).

### Differences in critical thinking, professionalism, leadership, and communication by nursing programs and academic years

From assessing the differences in critical thinking, professionalism, leadership, and communication according to the nursing programs and academic years (Table 3), it was found that the mean score of BS students for critical thinking was 85.7, 0.9 higher than the 84.8 for AD students. However, there was no statistically significant difference between the BS programs and three-year AD programs (t=1.017, P=0.310). The ranking of the mean scores by academic year of the BS students showed seniors with the highest scores (88.2), followed by freshmen (85.5), juniors (85.3) and lastly, sophomores (84.0). There were no statistically significant differences among the different years (F=2.446, P=0.065). Regarding three-year AD students, the junior scores were the highest at 86.3, while sophomore and freshmen scores were 84.8 and 83.2, respectively. There were no statistically significant differences among the different years either (F=1.654, P=0.194).

The mean score of the BS students for professionalism was 68.3, 0.1 higher than the 68.2 for AD students, but there was no statistically significant difference between the two groups (t=1.904, P=0.152). The mean score by academic year for BS students showed that seniors had the highest score (70.8), while freshmen and junior scores were 68.5 and 68.1, respectively. Sophomore scores were the lowest (65.7), indicating a statistically significant differences among the academic years (F=3.255, P=0.022). Using Tukey's post hoc test significant differences between seniors and sophomores were found as well. The mean score by academic year of the AD students was highest for freshman (69.5), while junior and sophomore scores were 68.8 and 66.8, respectively. However, there were no statistically significant differences among class years (F=1.904, P=0.152).

The mean score of the BS students for leadership was 150.8, 3.7 higher than the 147.1 for AD students, and it revealed a statistically significant difference between the two groups (t=2.272, P=0.024). The mean score by academic year of the BS students was highest for seniors (155.3), while freshmen and junior scores were 153.2 and 148.3, respectively. The sophomore score was the lowest (146.3). Furthermore, there were statistically significant differences among the academic years (F=3.733, P=0.012). Significant differences between seniors and sophomores were found as well using Tukey's post hoc test. The mean score per year of the AD students was highest for juniors (148.0), while sophomore and freshman scores were 147.7 and 145.3, respectively, but there were no statistically significant differences among class years (F=0.467, P=0.627).

The mean score of the AD students for communication was 87.3, 3.6 higher than the 83.7 for BS students, and it indicated statistically a significant difference between two groups (t=-4.175, P<0.001). The mean score by academic year of the BS students was highest for seniors (86.2), while junior and freshman scores were 83.5 and 83.2, respectively. The sophomore scores were the lowest (81.6). However, these scores revealed statistically significant differences among the academic years (F=3.052, P=0.029). In Tukey's post hoc test, significant differences between senior and sophomore years were found as well. The mean score per year of the AD students was highest for juniors (87.8), while sophomore and freshman scores were 97.5 and 86.3, respectively. There were no statistically significant differences among class years (F=0.381, P=0.684).

Results comparing the average scores focused on the last year of the two types of degree programs (Table 4). For critical thinking the mean score of BS seniors was 88.2, 1.9 higher than the 86.3 for three-year AD juniors. However, there was no statistically significant difference between the two groups (t=1.135, P=0.259). In terms of professionalism, the mean score of BS seniors was 70.7, 1.9 higher than the 68.8 for three-year AD juniors, but there was no statistically significant difference between the two groups (t=1.393, P=0.166). For leadership, the mean score of BS seniors was 155.3, 7.3 higher than the 148.0 for three-year AD juniors. This indicated statistically a significant difference between the two groups (t=2.540, P=0.012). For communication, the mean score of the three-year AD juniors was 87.8, 1.6 higher than the 86.2 for BS seniors, but this difference was not statistically significant (t=1.112, P=0.268).

## DISCUSSION

### Critical thinking

The average score on the CTDI in this study was lower than the study of Oh et al. [11] using the same tool, which was 102.3 for RN-BSN students and 103.7 for graduates. One explanation for this result could be that students and graduates from RN-BSN programs are expected to acquire a more critical orientation through professional nursing activities.

Regarding the differences in nursing programs in this study, BS students showed a higher disposition toward critical than the AD students, but it was not statistically significant. However, compared to the study of Cho [13], and that of Bae et al. [14] on three-year AD students using the same tool, the mean score for AD students in the previous study was little higher than that of the BS students in the current study. More detailed research on critical thinking is needed because while education for critical thinking has been emphasized in 4-year nursing education, it is unclear whether the curriculum is making a measureable difference. Considering differences by class level, both BS students and AD students revealed statistically significant differences by year in Yoon's study [7]. However, neither AD nor BS student mean scores revealed statistically significant differences by academic year in this study. Those in the last year of each programs attained higher scores. Therefore, a critical thinking disposition can apparently developed as nursing students advance through school. Also, in the case of BS programs, it is probable that freshmen attained higher critical thinking disposition scores than sophomore because of exposure to subjects such as philosophy and introduction to nursing. This is supported by the study results of Yang and Jung [15] which revealed that taking philosophy courses resulted in statistically significant different critical thinking disposition scores. Using Turkey's post hoc test as well, that the critical thinking disposition showed no differences by class year is a similar result to that of the study of Yang and Jung [15]. It is possible that encouraging students to choose relevant liberal arts courses and designing a nursing education curriculum with the principles of sequence, continuity, and integration will lead to the development of critical thinking skills and a critical disposition. The critical thinking process is interactively operated cognitive skills and affective skills including attitude and disposition traits. Therefore, Facione explained that critical thinking appears by interactions between critical thinking skills and disposition, so in order to measure critical thinking, both skill and disposition should be measured [16]. The study of Shin et al. [17] showed that there was a significant correlation between a disposition toward critical thinking and critical thinking skills in Korea. Research related to critical thinking in nursing has focused on definition [18,19], and, more recently, on teaching strategies [20,21]. However, the mutual understanding among nursing scholars of its use in educational outcomes lacks depth. Considering all these results, the experiences of learning to have a disposition toward critical thinking in nursing education needs to be analyzed in detail, and critical thinking skill measurement and comparison is required as well.

### Professionalism

Professionalism is a basic requirement for a Registered Nurse, so it is natural that there is no difference between BS and AD nursing programs in this study. For example, the two types of programs would provide similar education in professional ethics and the core values of nursing. However, compared to the studies by Oh et al. [11] and Kim et al. [9] on educational outcomes of RN-BSN students using the same tool, students in the present study attained lower scores than RN-BSN students and graduates of the other studies. This result could be due to the idea that students and graduates from RN-BSN programs are expected to acquire deeper professional values and attitudes through professional nursing activities in various health care settings. The score on professionalism in BS programs in this study increases as students progress through their four years of school. Students' learning experiences related professionalism in nursing education and practices need to be identified by academic year in the future because professionalism is increased as students continue to learn. Professionalism gives a framework for the criteria of nursing activity and a guide for the evaluation of nurses' behavior [22]. Therefore, research on professionalism showed that it was related to job satisfaction, organizational commitment, and professional self-concept [23,24]. It is indeed meaningful to measure professionalism as one of the outcome indicators of nursing education.

### Leadership

The mean leadership score of all subjects was 149.2 out of a possible of 200, and by nursing programs, BS students scored higher than AD students. The score of seniors was the highest, followed by that of the freshmen, which in turn was higher than the scores of sophomores and juniors. This positive change reflected a major nursing educational goal and curriculum of BS programs in training leaders to contribute to society. However, compared to the study of Oh et al. [11] using the same tool and researching RN-BSN students, students of this current study attained lower scores than the RN-BSN students (154.7) and graduates (160.8) of the other study. Leadership behaviors tended to increase in the later academic years both in three-year AD programs and BS programs. Through deeper theoretical and practical education in major nursing subjects, the importance of leadership is also emphasized. In addition, in comparison with the results of Oh et al. [11], the students' leadership scores were higher than graduates, indicating that leadership in nursing students is increasingly strengthened by learning. Therefore, in order to fully display leaders' capacities at work sites after graduation, leadership education should be consistently provided. On the other hand, the instrument used in this study measured a self-assessment of leadership traits, and was utilized as a tool for integrating components of outcome measurement necessary to leadership development. However, from the viewpoint of leadership theory this instrument focused on behavior theory as opposed to broadly including other sciences, so there are limits to its comprehensiveness. Leadership skills are needed that emphasize ethical and critical decision-making, initiating and maintaining effective working relationships, using mutually respectful communication and collaboration within inter-professional teams, care coordination, delegation, and developing conflict resolution strategies [25]. Leadership in the nursing profession is important for students at diverse levels, from preparing for the role of nursing leadership to eventually being the leaders in nursing practice.

### Communication

The mean communication score in this study (85.2 out of 120) was higher than the average score for this instrument. The score of the AD students was significantly higher than that of the BS students. This is because freshman in three-year AD programs begin clinical practice during their first year, which provides learning by practical experiences. By class year, the score of seniors (86.2) was the highest, followed by that of the freshman, which in turn was higher than the score of sophomore and juniors in BS programs. The score of seniors (87.8) was higher than that of the other students, revealing that communication gradually improved as a result of learning by academic years in three-year AD programs. However, compared to the study of Oh et al. [11], scores from this study were lower when compared to RN-BSN students (88.9) and graduates (90.1) measured by the same tool. Subsequently, a detailed analysis regarding communication content and experiences in nursing education is needed. Effective communication among health professionals is imperative to providing client-centered care, and is an essential professional competency that is conceptualized and developed during undergraduate education [26]. Communication skills of nursing students can be measured as a core indicator of final educational outcomes.

There are some limitations to this study. The sampling by convenience may limit generalization of the findings. Another limitation is that only four core concepts were delineated, and there was no instrument for appropriately measuring students' ability to think critically.

This study investigated the levels of critical thinking, professionalism, leadership, and communication of nursing students as outcome indicators of nursing education, and compared and analyzed the differences by nursing programs and academic years. Nursing students in BS programs and three-year AD programs were sampled using the convenience method and a total of 454 surveys were analyzed. Data collection was performed with four instruments: the CDTI, NSDF, SALI, and SCI. All scores for critical thinking disposition, professionalism, leadership, and communication of nursing students were slightly above the average scores for these instruments. There were statistically significant differences in the BS program and three-year AD programs leadership and communication scores. This indicated that leadership development in BS programs was greater than in three-year AD programs. Conversely, in communication, three-year AD students' scores were higher than those of BS students. The mean scores for leadership, communication, and professionalism tended to increase as students progressed through the program, while scores of critical thinking disposition did not. Based on these findings, recommendations are as follows:

1. Properties of the nursing educational outcomes must be defined.

2. Standardized instruments for research replication and verification are needed.

## Figures and Tables

**Table 1:**
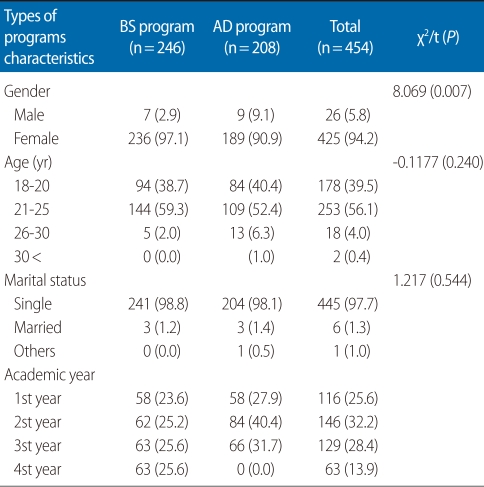
General characteristics of the participants

Values are presented as number (%). BS, baccalaureate; AD, associate degree.

**Table 2:**
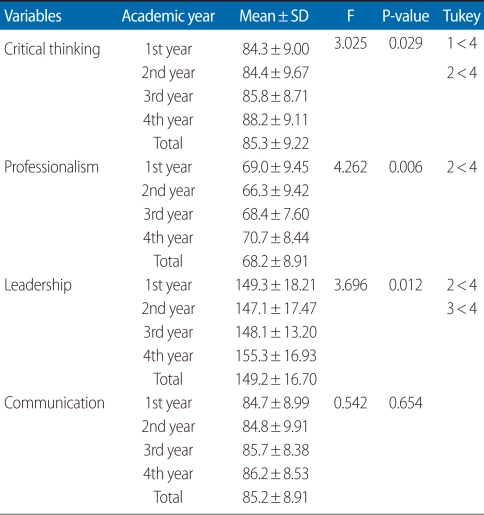
Mean scores for critical thinking, professionalism, leadership, and communication (n=454)

BS, baccalaureate; AD, associate degree.

**Table 3:**
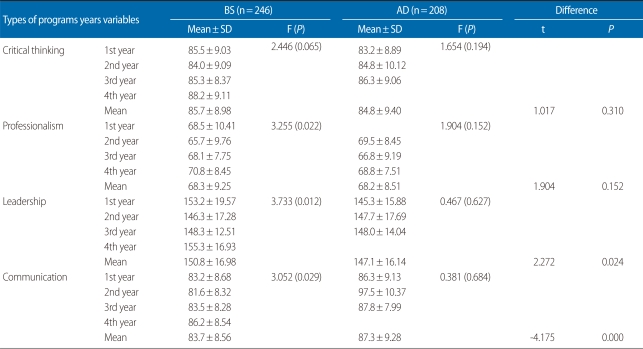
Mean scores for each variable by nursing program and academic year (n=454)

**Table 4:**
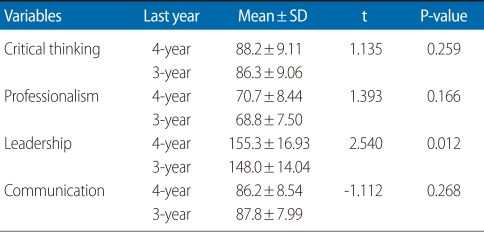
Mean scores for each variable in last year of nursing program (n=129)
